# Perceived aging risk and private medical insurance intentions: education, subjective health, and a China–Malaysia comparison

**DOI:** 10.3389/fpubh.2026.1731431

**Published:** 2026-06-04

**Authors:** Zhangwei Zheng, Hafizuddin-Syah B.A.M, Hafizah Omar Zaki, Qin Lingda Tan

**Affiliations:** 1 Sunway Business School, Sunway University, Sunway, Selangor, Malaysia; 2 Faculty of Economics and Management, Universiti Kebangsaan Malaysia, Bangi, Selangor, Malaysia

**Keywords:** cross-cultural, education, insurance purchase intention, population aging, private medical insurance, subjective health

## Abstract

**Introduction:**

Population aging raises uncertainty about future healthcare costs, pension adequacy, and financial security, yet evidence remains limited on how aging-related risk perceptions shape private medical insurance (PMI) intentions among adult consumers aged 18–64. This study examines whether perceived aging risk (AR) is associated with PMI purchase intentions and whether education, subjective health status, and national context condition this association.

**Methods:**

Survey data from consumers aged 18–64 in China (*N* = 484) and Malaysia (*N* = 415) were analyzed using PLS-SEM. Two country-specific models and one pooled boundary-condition model (*N* = 899) were estimated, with demographic, health, insurance-experience, and income controls.

**Results:**

AR was consistently and positively associated with PMI purchase intentions across all models, whereas education and subjective health status showed no direct effects. In Malaysia, the AR–intention association was stronger among lower-education respondents and those reporting better subjective health; these moderation effects were not observed in China. The pooled model indicated that national context conditioned only education-based moderation.

**Discussion:**

The findings suggest that AR is a robust expectancy-based correlate of PMI intentions, but its translation into intention depends on context-specific consumer resources. PMI communication in aging societies should combine aging-risk framing with locally calibrated education-sensitive implementation.

## 1 Introduction

Perceptions of future uncertainty and economic risk significantly shape consumers’ financial decisions, notably regarding insurance adoption. Existing literature suggests that perceived future economic and health-related risks increase consumers’ motivation to engage in precautionary financial behaviors, such as insurance purchasing (1, 2). In rapidly aging societies, demographic aging itself becomes a salient source of future-oriented uncertainty because it raises concerns about personally consequential later-life vulnerability. In this study, perceived aging risk (AR) refers to consumers’ perceived uncertainties and concerns associated with demographic aging, including anticipated pressures related to healthcare costs, pension adequacy, and financial security (3). Importantly, AR is not reducible to current health risk or a transaction-specific perceived risk; rather, it captures an aging-contextualized appraisal that can make later-life protection decisions salient in the present. Although it is reasonable to expect that greater perceptions of aging risk will stimulate demand for private medical insurance (PMI), empirical evidence at the individual level remains limited, particularly regarding young populations (4, 5).

More importantly, prior studies on aging and insurance purchase have largely treated consumers as homogeneous, overlooking how demographic characteristics might influence individuals’ responses to aging risk (4, 6, 7). This omission matters because the young adults constitute a critical decision window in which aging-related concerns may already shape preventive planning, yet evidence on how AR is translated into PMI intentions remains scarce. Two critical yet understudied demographic factors that may shape the AR–insurance relationship are education level (EDU) and subjective health status (SHS). While previous research suggests that higher EDU may equip individuals with enhanced cognitive resources and long-term planning capabilities (8, 9), empirical findings regarding its actual influence on insurance intentions remain mixed. Similarly, SHS—reflecting perceived health vulnerability—may either amplify or dampen individuals’ responsiveness to AR by shaping whether aging-related risk is perceived as urgent, manageable, or feasible to address through PMI (10, 11). Accordingly, the core gap is not only whether AR predicts PMI intentions, but when and for whom AR becomes motivationally decisive enough to trigger preventive insurance planning.

To address these gaps, this study investigates how EDU and SHS moderate the relationship between AR and PMI purchase intentions among adult consumers aged 18–64. Expectancy theory (12) provides the primary theoretical anchor: individuals are more likely to consider PMI when aging-related adverse outcomes are perceived as plausible, PMI is viewed as instrumental for mitigating such exposure, and the anticipated consequences carry negative valence. Building on this logic, the study adopts a resource-based account of heterogeneity, in which education and subjective health represent individual conditions that may shape whether aging-related risk perceptions become actionable insurance intentions.

The study further examines these relationships in China and Malaysia, two developing countries undergoing rapid population aging but characterized by distinct healthcare, insurance, and public–private risk-sharing arrangements (13, 14). This comparison is theoretically useful because national context may shape the perceived instrumentality and substitutability of PMI. Public provision, private insurance development, and culturally patterned expectations of risk sharing may affect whether consumers regard PMI as a necessary supplement to existing protection. Accordingly, national context is examined as a boundary condition for the AR–PI association and for the moderating roles of EDU and SHS.

This research contributes to the consumer behavior and health insurance literature in three ways. First, it advances research on precautionary financial behavior by conceptualizing AR as an aging-contextualized, future-oriented risk appraisal and examining its relevance among consumers aged 18–64. Second, it extends expectancy-based explanations of PMI intention by showing that AR is not translated into purchase intention uniformly; rather, this association is conditioned by education and subjective health. Third, by comparing China and Malaysia, the study identifies national context as a boundary condition in aging-risk-related insurance planning and provides implications for PMI communication, consumer segmentation, and public–private insurance coordination.

## 2 Literature review and hypothesis development

### 2.1 Aging risk and insurance purchase intentions

Risk perception fundamentally shapes consumer decisions, particularly in contexts involving financial uncertainty such as insurance adoption (1, 2). AR, defined as perceived uncertainties associated with demographic aging—including concerns about future healthcare expenses and economic insecurity—has become particularly salient due to rapid population aging globally (4). Prior research highlights that perceptions of increased healthcare costs and declining pension adequacy encourage precautionary financial behaviors, including PMI adoption (15, 16). However, despite the evident connection, studies explicitly examining how aging-related uncertainties influence young consumers’ PMI purchase intentions remain limited, with much research instead focusing on older adults (7, 17).

Drawing on expectancy theory (12), which suggests that individuals are motivated to engage in behaviors they believe can help mitigate anticipated future risks (18, 19), heightened AR perceptions are expected to be positively associated with PMI purchase intentions. Thus:

*H1:* AR is positively associated with PI.

### 2.2 Direct influence of education level and subjective health status

EDU and SHS are critical demographic variables shaping consumer behavior, particularly in insurance decisions (20, 21). EDU significantly influences financial literacy, cognitive capability, and individuals’ ability to plan for future financial risks, thereby affecting insurance adoption (22, 23). Empirical evidence largely supports the positive relationship between higher education levels and greater insurance uptake, attributed primarily to superior risk assessment and financial planning skills (23, 24). Conversely, lower-educated consumers may exhibit weaker long-term financial planning capabilities and limited understanding of insurance benefits, reducing their likelihood of insurance adoption (11). Accordingly:

*H2a:* EDU is significantly associated with PI.

SHS, or self-rated health, captures individuals’ subjective assessment of their own health conditions (25). Individuals who perceive their health negatively are generally more motivated to secure insurance due to increased anticipation of healthcare needs (26). Conversely, healthier individuals typically perceive lower healthcare risks and thus exhibit weaker intentions to purchase PMI (21). This aligns with prior empirical findings highlighting the inverse relationship between better SHS and insurance adoption (20). Hence:

*H2b:* SHS is significantly associated with PI.

### 2.3 Moderating effects: education and subjective health as resource-based boundary conditions

The association between AR and PMI purchase intentions is unlikely to be uniform across individuals. Expectancy theory explains why aging-related uncertainty may be associated with insurance planning, but it does not by itself explain why some consumers translate the same risk appraisal into stronger purchase intentions than others. EDU and SHS may shape this translation because they reflect different individual resources and constraints relevant to future-oriented decision-making.

Education is closely associated with cognitive resources, financial literacy, information-search capability, and long-term planning capacity (9, 23). One pathway suggests that higher EDU strengthens proactive planning and risk appraisal, thereby amplifying the extent to which AR is translated into PMI intentions. A competing pathway suggests that higher-EDU consumers may have broader access to alternative planning instruments and risk-management options, which can reduce the marginal instrumentality of PMI as the primary response to AR. Conversely, lower-EDU consumers may face more constrained planning options, making PMI a more salient and actionable safeguard when aging-related risk becomes salient. Therefore:

*H3a:* EDU significantly moderates the relationship between AR and PI.

SHS may also condition the AR–PI relationship because self-rated health captures perceived health vulnerability and perceived capacity to engage in preventive planning (10, 11). One pathway suggests that poorer self-rated health heightens perceived vulnerability and strengthens responsiveness to aging-related uncertainty. A competing pathway suggests that better self-rated health may increase perceived feasibility and readiness to engage in preventive planning, thereby strengthening the translation of AR into PMI intentions. Hence:

*H3b:* SHS significantly moderates the relationship between AR and PI.

### 2.4 National context as a boundary condition

The AR–PI association and its demographic contingencies may depend on national contexts with different healthcare arrangements, insurance-market structures, and expectations of public–private risk sharing (13, 14). China and Malaysia are both developing countries facing population aging, but they differ in the organization of public healthcare, the role of supplementary private coverage, and the perceived responsibilities of state, family, and market institutions in managing later-life risk (15, 27–29). These differences may shape how consumers evaluate PMI as an instrumental response to aging-related uncertainty.

National context may condition the AR–PI association in two ways. First, the perceived instrumentality of PMI may vary depending on whether consumers view private coverage as essential, supplementary, or substitutable relative to public protection. Second, the roles of EDU and SHS may vary because education-related planning resources, health perceptions, and perceived actionability can operate differently across institutional environments. Accordingly, this study proposes:

*H4a:* National context significantly conditions the association between AR and PI.

*H4b:* National context significantly conditions the moderating role of EDU in the AR–PI association.

*H4c:* National context significantly conditions the moderating role of SHS in the AR–PI association.

### 2.5 Conceptual framework

**Figure 1:**
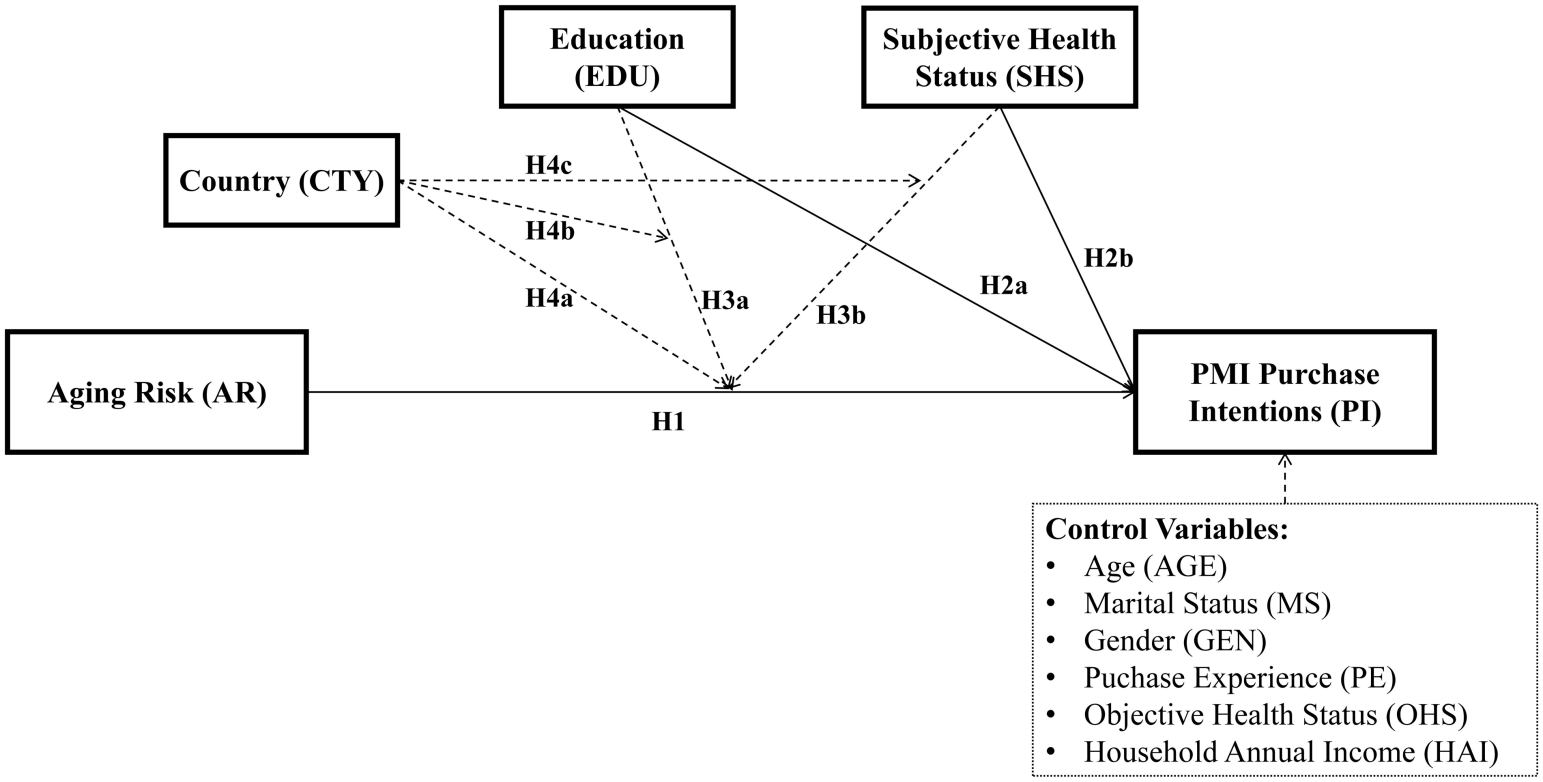
Conceptual framework.

Based on the theoretical foundations discussed above, this study proposes a conceptual framework encompassing three interrelated analyses. Studies 1 and 2 examine the AR–PI association and the moderating roles of EDU and SHS within China and Malaysia, respectively (H1–H3). Study 3 pools the two national samples to examine whether national context further conditions the AR–PI association and its demographic contingencies (H4a–H4c). To reduce confounding, the analysis includes age (AGE), gender (GEN), marital status (MS), purchase experience (PE), objective health status (OHS), and household annual income (HAI) as control variables for PMI purchase intentions. The complete conceptual model is illustrated (Figure 1).

## 3 Methodology

### 3.1 Research design and sampling procedures

This study adopts a cross-sectional, multi-study design comprising two country-specific analyses and one pooled boundary-condition analysis. Study 1 and Study 2 independently examine the proposed model within China and Malaysia, respectively. Study 3 pools the two datasets to test whether national context further conditions the observed AR–PI association and its demographic contingencies. In the pooled model, CTY is coded as a binary contextual variable (Malaysia = 0, China = 1) and is used to construct the relevant interaction terms.

Measurement invariance across the two country groups was assessed using MICOM (Appendix A). Because partial measurement invariance was not established for several key constructs, Study 3 is not treated as a multi-group analysis (MGA)-style comparison of country-specific latent path coefficients. Instead, it is interpreted as a pooled boundary-condition analysis that examines whether national context is associated with systematic differences in the observed model relationships. This positioning preserves the contextual purpose of the China–Malaysia comparison while delimiting the inferential scope of country-related interaction effects.

Data were collected using online questionnaires administered separately to young consumers (aged 18–64), in accordance with the United Nations’ aging classification standard (30). Respondents were selected via purposive sampling based on three criteria: age eligibility, local residency, and eligibility for PMI coverage. To ensure that respondents had meaningful exposure to and access to PMI products, data collection targeted young consumers in major metropolitan areas with relatively developed insurance markets. The sample sizes for both countries surpassed the minimum recommended threshold of 384 participants (31). Specifically, Study 1 collected data from Shenzhen and Shanghai (China) between March 15–31, 2024 (*n* = 484), while Study 2 was conducted in Kuala Lumpur (Malaysia) from March to August 2024 (*n* = 415). Study 3 combined these datasets (*N* = 899) for the pooled boundary-condition analysis. Detailed demographic characteristics are presented in Tables 1, 2.

**Table 1:** Demographic information of respondents in Study 1 (China).

Demographic items	Options	*N* (484)	%
Age Group	18–24	73	15.08%
	25–54	307	63.43%
	55–64	104	21.49%
Gender	Male	239	49.38%
	Female	245	50.62%
Education level	Postgraduate	67	13.84%
	Undergraduate	288	59.50%
	College	66	13.64%
	High School	58	11.98%
	Less than High School	5	1.03%
Marital status	Single	113	23.35%
	Married	371	76.65%
Purchase experience	Yes	310	64.05%
	No	174	35.95%
Household annual income	Less than ¥200,000	194	40.08%
	¥200,001–¥300,000	134	27.69%
	¥300,001 or more	156	32.23%
Subjective health status	Excellent	59	12.19%
	Good	269	55.58%
	Fair	138	28.51%
	Poor	18	3.72%
Objective health status (chronic disease)	Yes	179	36.98%
	No	305	63.02%

**Table 2:** Demographic information of respondents in Study 2 (Malaysia).

Demographic items	Options	*N* (415)	%
Age Group	18–24	10	2.41%
	25–54	255	61.45%
	55–64	150	36.14%
Gender	Male	155	37.35%
	Female	260	62.65%
Education level	Postgraduate	89	21.45%
	Undergraduate	186	44.82%
	College	94	22.65%
	High School	45	10.84%
	Less than High School	1	0.24%
Marital status	Single	289	69.64%
	Married	126	30.36%
Purchase experience	Yes	319	76.87%
	No	96	23.13%
Household annual income	B40	166	40.00%
	M40	120	28.92%
	T20	129	31.08%
Subjective health status	Excellent	85	20.48%
	Good	207	49.88%
	Fair	115	27.71%
	Poor	8	1.93%
Objective health status (chronic disease)	Yes	61	14.70%
	No	354	85.30%

### 3.2 Measures

The primary constructs, AR and PI, were measured using established multi-item scales from prior research (Appendix B). AR, defined as consumers’ perceptions of uncertainties associated with aging-related healthcare costs, pension adequacy, and financial security, was measured using a scale adopted from Zheng et al. (3), which was originally adapted from Jacoby and Kaplan (32), Ilgen et al. (33), Pintrich and De Groot (34), Brahmana et al. (1), and Amali et al. (35). PI was directly measured using items adopted from Ajzen (36) and Hasan and Rahman (37). Both constructs employed seven-point Likert scales (1 = strongly disagree; 7 = strongly agree).

EDU and SHS were captured as demographic moderators through single-item self-report scales, reflecting respondents’ educational attainment (five-point scale ranging from “less than high school” to “postgraduate”) and subjective health perceptions (four-point scale from “poor” to “excellent”). To isolate the hypothesized relationships from confounding influences, six control variables were included: age (AGE; continuous), gender (GEN; binary), marital status (MS; binary), purchase experience (PE; binary), objective health status (OHS; binary, indicating medically diagnosed chronic diseases), and household annual income (HAI; three-level country-specific income-position indicator). Higher HAI values indicate higher household income within each national context.

All survey items were originally developed in English and subsequently translated into Chinese through a rigorous back-translation procedure to ensure semantic equivalence, accuracy, and cultural appropriateness. The final questionnaire was presented bilingually in English and Chinese.

### 3.3 Measurement validation procedures

Before full-scale data collection, the survey underwent a structured two-phase validation process. First, a content validity assessment was conducted with an expert panel (two academics specializing in consumer behavior, an insurance industry professional, and a policymaker) via online consultations. Feedback led to refinements of construct definitions, wording clarity, and contextual relevance for both national samples. Subsequently, a pilot test (*n* = 57; March 8–10, 2024) was conducted to evaluate psychometric properties. Preliminary partial least squares structural equation modeling (PLS-SEM) analyses confirmed acceptable levels of internal consistency, convergent validity, and discriminant validity according to established thresholds (38, 39), verifying readiness for primary data collection.

Additionally, to mitigate common method bias, the survey emphasized anonymity and confidentiality, and collinearity diagnostics indicated no serious concern.

### 3.4 Analytical strategy

PLS-SEM was employed because it is well suited for prediction-oriented models with interaction effects, performs robustly under non-normal data, and is appropriate for moderate sample sizes in exploratory theory development. Accordingly, the data analysis was executed using SmartPLS 4.0 with a two-step PLS-SEM analytical procedure recommended by Hair et al. (40). Initially, the psychometric robustness of the measurement model was assessed by evaluating indicator reliability (factor loadings ≥ 0.708), internal consistency (Cronbach’s alpha and composite reliability, CR ≥ 0.7), convergent validity (average variance extracted, AVE ≥ 0.5), and discriminant validity using the Fornell-Larcker criterion and the heterotrait-monotrait ratio (HTMT ≤ 0.9), following the guidelines of Hair et al. (38, 39).

In the subsequent structural model analysis, collinearity was assessed through Variance Inflation Factor (VIF), ensuring values remained below the critical threshold of 3 (38, 39). Model explanatory capacity was evaluated using coefficient of determination (R^2^) and effect sizes (f^2^), following Cohen (41) and Henseler et al. (42) (f^2^: 0.02/0.15/0.35 = small/medium/large; R^2^: 0.25/0.50/0.75 = weak/moderate/substantial). Predictive accuracy was tested using the Cross-Validated Predictive Ability Test (CVPAT), following Liengaard et al. (43) and Sharma et al. (44). Hypotheses were statistically examined through bootstrapped path coefficient estimates [5,000 resamples; (40)].

The analysis also incorporated moderation effects. In Studies 1 and 2, EDU and SHS were examined as moderators of the AR–PI relationship by including AR × EDU and AR × SHS interaction terms in the country-specific structural models. In Study 3, CTY × AR, CTY × EDU × AR, and CTY × SHS × AR were included to test whether national context further conditioned the observed relationships in the pooled model. Given the MICOM results, these country-related interaction effects are interpreted as pooled boundary-condition evidence rather than formal MGA-style comparisons of country-specific latent path coefficients. All structural models include AGE, GEN, MS, PE, OHS, and HAI as control variables.

### 3.5 Ethical considerations

This research strictly adhered to established ethical guidelines. Approval was obtained from The National University of Malaysia Research Ethics Committee (reference no. JEP-2024-001, dated March 8, 2024). Participants were informed explicitly in the questionnaire instructions that participation was voluntary, anonymous, and confidential, with survey completion implying informed consent. Personal identifiers were removed prior to data analysis to protect participant confidentiality, conforming to ethical research standards (45).

## 4 Results

### 4.1 Measurement model assessment

The measurement model was evaluated in terms of indicator reliability, internal consistency, convergent validity, and discriminant validity, following established guidelines (38, 39). Across all three studies, the factor loadings for all items measuring AR and PI exceeded the recommended threshold of 0.708 (Table 3), indicating that each indicator reliably reflects its intended latent construct. Cross-loadings for all indicators across the three studies are reported in Appendix C to provide additional item-level evidence of measurement quality.

**Table 3:** Measurement items.

Variable	Items	Study 1		Study 2		Study 3	
		Loadings	Outer VIF	Loadings	Outer VIF	Loadings	Outer VIF
Aging risk (AR)	AR1	0.737	1.823	0.782	1.968	0.793	2.132
	AR2	0.817	2.363	0.869	3.292	0.862	3.034
	AR3	0.808	2.218	0.827	2.916	0.846	2.777
	AR4	0.814	2.29	0.833	3.222	0.849	2.979
	AR5	0.816	2.315	0.821	2.903	0.84	2.797
	AR6	0.796	2.15	0.841	3.452	0.847	2.947
	AR7	0.795	2.108	0.847	3.65	0.854	2.936
	AR8	0.743	1.777	0.799	2.743	0.79	2.249
PMI purchase intentions (PI)	PI1	0.862	2.83	0.878	3.103	0.892	3.41
	PI2	0.867	2.87	0.86	2.797	0.886	3.314
	PI3	0.86	2.803	0.852	2.646	0.882	3.194
	PI4	0.862	2.772	0.847	2.645	0.884	3.219
	PI5	0.838	2.427	0.852	2.575	0.864	2.830
	PI6	0.869	2.973	0.858	2.743	0.871	2.981

Internal consistency was supported by Cronbach’s alpha and CR values (Table 4), which fell within the acceptable range of 0.70 to 0.95 for all constructs in Study 1 and Study 2, suggesting strong internal consistency without redundancy concerns. In Study 3, the CR for PI slightly exceeded the 0.95 guideline (CR = 0.954); however, this was deemed acceptable given consistently high loadings, robust convergent validity (AVE = 0.774), the absence of comparable inflation in the country-specific models, and indicator-level (outer) VIF values that did not indicate problematic redundancy (Table 3).

**Table 4:** Measurement indicators.

Variable	Study	Cronbach’s alpha	Composite reliability (CR)	Average variance extracted (AVE)
Aging risk (AR)	Study 1	0.914	0.930	0.626
	Study 2	0.934	0.946	0.685
	Study 3	0.938	0.949	0.698
PMI purchase intentions (PI)	Study 1	0.929	0.944	0.739
	Study 2	0.928	0.944	0.736
	Study 3	0.942	0.954	0.774

All constructs demonstrated adequate convergent validity, with AVE values exceeding the 0.50 benchmark in every study (Table 4), indicating that each construct captured a sufficient proportion of variance from its indicators.

Discriminant validity was confirmed through both the Fornell-Larcker criterion and the HTMT (Table 5). The square roots of AVEs were greater than the corresponding inter-construct correlations in all cases, and HTMT values remained below 0.90, confirming that AR and PI were empirically distinct. These results collectively support the adequacy of the measurement model for subsequent structural analysis.

**Table 5:** Discriminant validity.

Study	Variables	Heterotrait-monotrait ratio (HTMT)		Fornell-Larcker criterion	
		AR	PI	AR	PI
Study 1	AR			0.791	
	PI	0.843		0.778	0.86
Study 2	AR			0.828	
	PI	0.797		0.752	0.858
Study 3	AR			0.836	
	PI	0.857		0.805	0.88

### 4.2 Structural model assessment

Prior to hypothesis testing, the structural model was evaluated in terms of collinearity, explanatory power, and predictive relevance to ensure the robustness of path estimations. Collinearity was examined using inner VIF values. As shown in Table 6, all inner VIF values fell below the commonly accepted threshold of 3.0 (39), indicating no evidence of multicollinearity that would distort regression estimates.

**Table 6:** Collinearity and explanatory power.

Study	Variable	R^2^	Path	f^2^	Inner VIF
Study 1	PI	0.653 (moderate)	AR→PI	1.324 (substantial)	1.118
Study 2	PI	0.588 (moderate)	AR→PI	1.242 (substantial)	1.087
Study 3	PI	0.68 (moderate)	AR→PI	0.716 (substantial)	2.460

The model’s explanatory capacity was assessed through R^2^ and f^2^ (Table 6). Across all three analyses, R^2^ values for PI ranged from 0.588 to 0.680, indicating moderate explanatory power (42, 46). The effect sizes associated with AR were substantial across models (f^2^ = 0.716–1.324) (41), underscoring the robustness of the AR–PI association after accounting for the specified control variables.

To evaluate the model’s predictive utility beyond the estimation sample, the CVPAT was employed. In all three studies, the prediction error of the PLS model was lower than that of the benchmark information-aware (IA) model (Table 7), supporting the model’s out-of-sample predictive relevance (43, 44).

**Table 7:** Predictive power: cross-validated predictive ability test (CVPAT).

Study	Variable	PLS loss	IA loss	Result
Study 1	PI	0.886	1.653	PLS loss < IA loss
Study 2	PI	1.352	2.266	PLS loss < IA loss
Study 3	PI	1.112	2.286	PLS loss < IA loss

### 4.3 Hypotheses testing and moderation effects

The structural model results indicate that AR is positively associated with PI across all three analyses, suggesting that heightened concerns about aging-related uncertainty are consistently linked to stronger PMI purchase intentions after accounting for the specified control variables (H1 supported). EDU and SHS did not exhibit significant direct effects on PI in any model (H2a and H2b not supported), suggesting that their relevance lies primarily in conditioning the AR–PI association rather than directly predicting PMI intentions. The moderation results are presented below for China, Malaysia, and the pooled boundary-condition model.

#### 4.3.1 Moderation effects in Study 1 (China)

**Figure 2:**
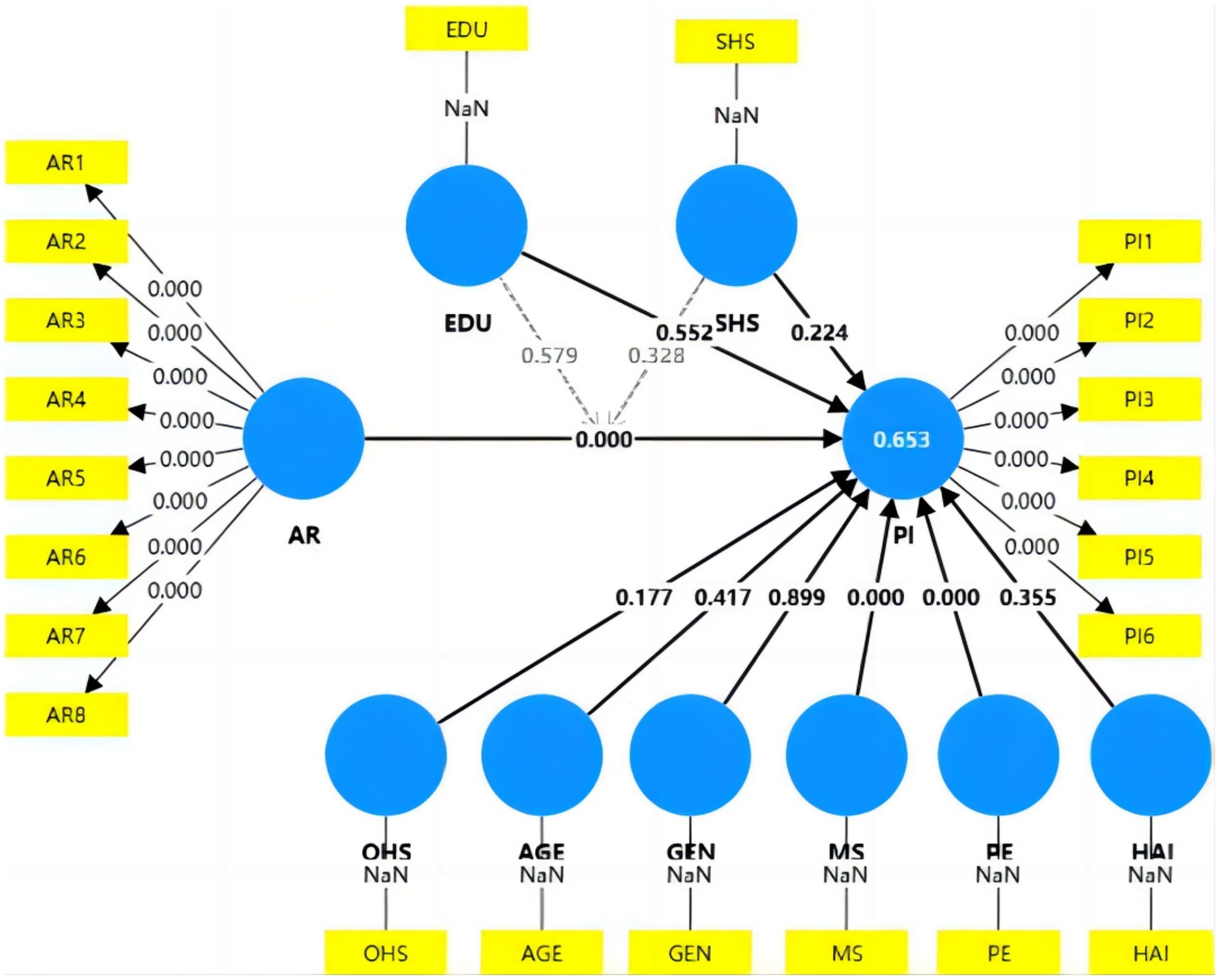
Structural model in Study 1.

In China, neither EDU × AR nor SHS × AR significantly moderated the AR–PI relationship after accounting for the specified control variables (H3a and H3b not supported), indicating that the positive association between AR and PI was relatively consistent across education and subjective health groups in this sample (Table 8; Figure 2). Thus, the China model provides evidence for the direct AR–PI association but not for education- or health-based moderation.

**Table 8:** Path coefficients and hypotheses test results [bootstrapping (*n* = 5,000)].

Study	Hypotheses	Path	Std Beta	Std Error	t	P	Confidence interval (95%) bias corrected	Decision
Study 1	H1	AR→PI	0.717	0.032	22.49	0.000	[0.648, 0.774]	Supported
	H2a	EDU→PI	0.021	0.036	0.594	0.552	[−0.046, 0.095]	Not supported
	H2b	SHS→PI	−0.036	0.03	1.216	0.224	[−0.094, 0.022]	Not supported
	H3a	EDU × AR→PI	0.014	0.026	0.556	0.579	[−0.035, 0.066]	Not supported
	H3b	SHS × AR→PI	0.03	0.03	0.979	0.328	[−0.031, 0.086]	Not supported
Study 2	H1	AR→PI	0.745	0.025	30.127	0.000	[0.691, 0.790]	Supported
	H2a	EDU→PI	0.037	0.042	0.892	0.372	[−0.047, 0.115]	Not supported
	H2b	SHS→PI	−0.017	0.036	0.474	0.636	[−0.091, 0.051]	Not supported
	H3a	EDU × AR→PI	−0.091	0.037	2.434	0.015	[−0.168, −0.020]	Supported
	H3b	SHS × AR→PI	0.115	0.036	3.222	0.001	[0.038, 0.182]	Supported
Study 3	H1	AR→PI	0.751	0.028	26.451	0.000	[0.693, 0.804]	Supported
	H2a	EDU→PI	0.019	0.037	0.509	0.611	[−0.058, 0.090]	Not supported
	H2b	SHS→PI	0.024	0.036	0.678	0.498	[−0.042, 0.100]	Not supported
	H3a	EDU × AR→PI	−0.097	0.037	2.647	0.008	[−0.174, −0.030]	Supported
	H3b	SHS × AR→PI	0.115	0.034	3.399	0.001	[0.048, 0.180]	Supported
	H4a	CTY × AR→PI	−0.036	0.045	0.803	0.422	[−0.124, 0.051]	Not supported
	H4b	CTY × EDU × AR→PI	0.109	0.046	2.386	0.017	[0.023, 0.205]	Supported
	H4c	CTY × SHS × AR→PI	−0.077	0.046	1.66	0.096	[−0.170, 0.014]	Not supported

AGE, GEN, MS, PE, OHS, and HAI were included as control variables in all structural models but are not tabulated for parsimony. H4a–H4c refer to pooled boundary-condition tests; the country-related interaction effects are not interpreted as MGA-style comparisons of country-specific latent path coefficients.

#### 4.3.2 Moderation effects in Study 2 (Malaysia)

**Figure 3:**
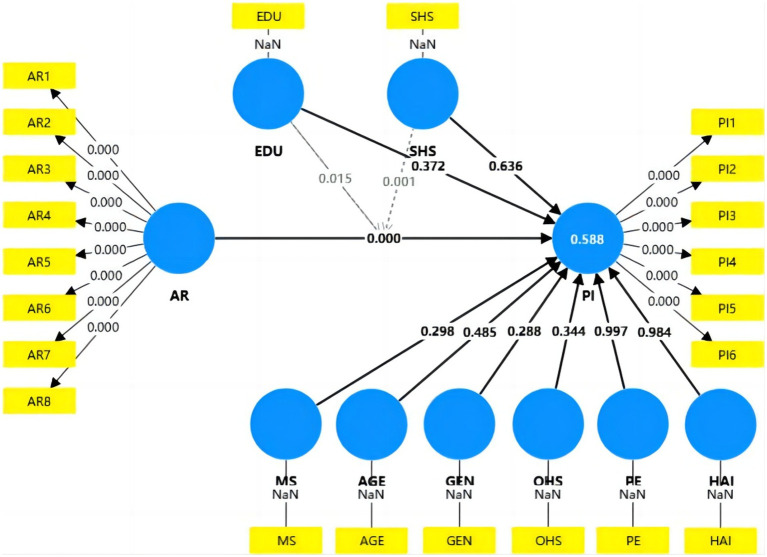
Structural model in Study 2.

**Figure 4:**
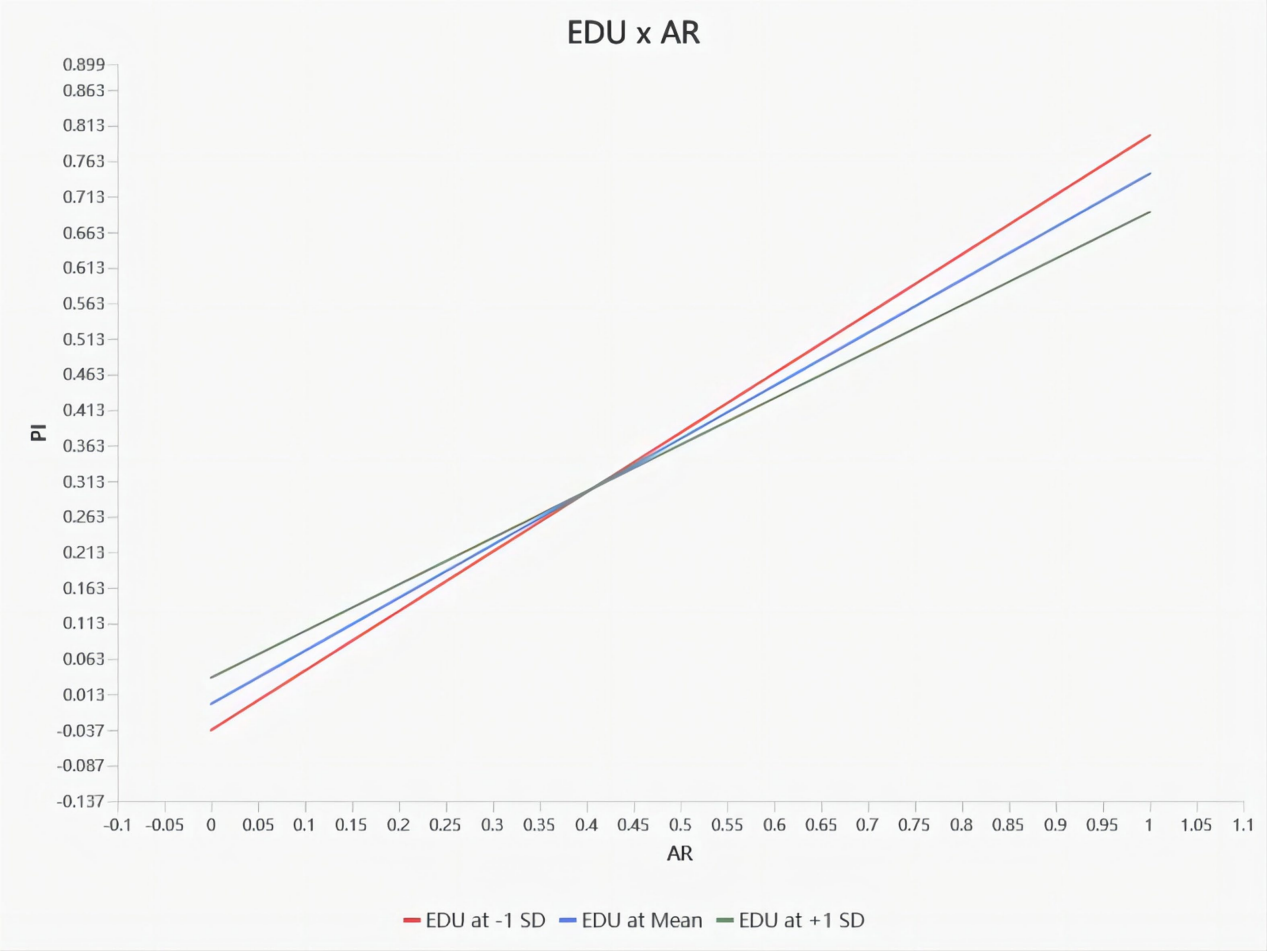
EDU simple slope analysis in Study 2.

**Figure 5:**
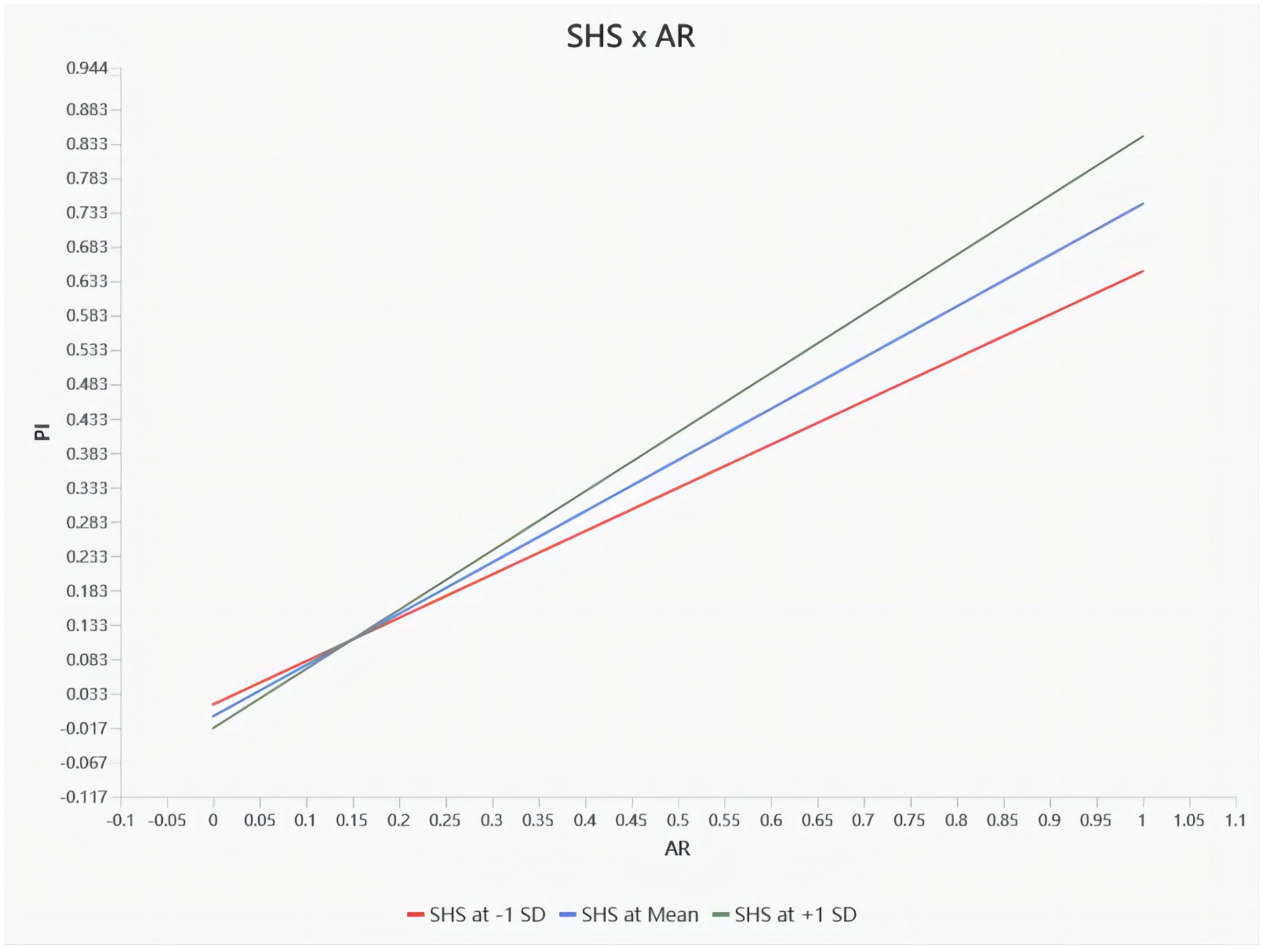
SHS simple slope analysis in Study 2.

In Malaysia, significant moderation effects emerged after accounting for the specified control variables (Table 8; Figure 3). EDU negatively moderated the AR–PI relationship (*β* = −0.091, *p* = 0.015; H3a supported), indicating that the positive association between AR and PI was stronger among lower-EDU respondents and weaker among higher-EDU respondents (Figure 4). SHS positively moderated the AR–PI relationship (β = 0.115, *p* = 0.001; H3b supported), indicating that respondents with better self-rated health showed a stronger association between AR and PI (Figure 5). These findings suggest that education- and health-related boundary conditions are more visible in the Malaysia sample than in the China sample.

#### 4.3.3 Study 3: pooled boundary-condition analysis

**Figure 6:**
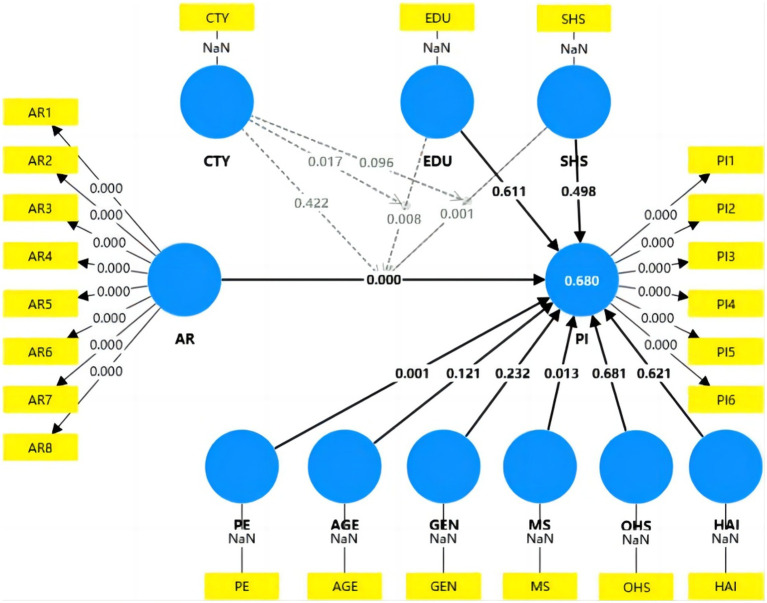
Pooled boundary-condition model in Study 3.

Study 3 examined whether national context further conditioned the observed model relationships in the pooled China–Malaysia sample (Table 8; Figure 6). Because MICOM did not establish partial measurement invariance for several key constructs (Appendix A), the pooled country-related interactions are interpreted as boundary-condition effects rather than MGA-style comparisons of country-specific latent path coefficients.

In the pooled model, CTY × AR was not significant (β = −0.036, *p* = 0.422; H4a not supported), indicating no clear evidence that national context conditioned the overall AR–PI association. CTY × EDU × AR was significant (β = 0.109, *p* = 0.017; H4b supported), indicating that the education-related moderation pattern varied by national context after accounting for the specified control variables. CTY × SHS × AR was not significant (β = −0.077, *p* = 0.096; H4c not supported), indicating no clear evidence that the SHS-related moderation pattern varied by national context. These results suggest that national context is more relevant to education-related heterogeneity than to the overall AR–PI association or SHS-related moderation.

## 5 Discussion and conclusion

### 5.1 Discussion of key findings

This study examines how perceived aging risk (AR) is associated with private medical insurance purchase intentions (PI) among adult consuumers aged 18-64 and how education (EDU), subjective health status (SHS), and national context condition this association. Overall, the findings identify AR as a robust correlate of PMI intentions and show that demographic and contextual contingencies shape the strength of this association.

AR is positively associated with PI across all three analyses (H1 supported), consistent with prior evidence that future-oriented risk perceptions are linked to precautionary financial behaviors such as insurance adoption (1, 2). The magnitude of this relationship is practically meaningful: the models explain a moderate portion of variance in PI (R^2^ = 0.588–0.680), and AR exhibits substantial effect sizes (f^2^ = 0.716–1.324). From an expectancy-based perspective, aging-related uncertainty may increase the perceived relevance of future losses and the perceived instrumentality of PMI as a risk-mitigation option. By contrast, EDU and SHS show no significant direct effects on PI (H2a and H2b not supported), suggesting that these demographic attributes function mainly as boundary conditions rather than uniform predictors of insurance intentions.

In Study 1 (China), neither EDU nor SHS significantly moderates the AR–PI relationship (H3a and H3b not supported), indicating broadly similar AR–PI associations across education and perceived health groups. One plausible interpretation is that when institutionalized risk sharing and expectations of public provision are salient, subgroup differences in education or self-rated health may exert weaker marginal influence on how consumers evaluate PMI. In such contexts, AR may operate as a shared future-oriented concern. This interpretation is consistent with research suggesting that institutional environments can shape the behavioral relevance of individual differences in insurance-related decision-making (4, 13, 14), although future studies should test this explanation using direct measures of public insurance reliance, perceived adequacy of public provision, and PMI substitutability.

In Study 2 (Malaysia), the AR–PI relationship is more heterogeneous. EDU negatively moderates the AR–PI association (H3a supported), indicating stronger AR responsiveness among lower-EDU respondents. This pattern should be interpreted as a demographic boundary effect rather than as evidence of a purely cognitive mechanism. Education may capture differences in financial literacy, information access, planning experience, and perceived reliance on PMI as an actionable safeguard. For lower-EDU consumers, PMI may represent a more salient and concrete form of protection when aging-related uncertainty becomes personally relevant. For higher-EDU consumers, broader planning options or alternative risk-management strategies may reduce the marginal role of PMI as the primary response to AR. The persistence of this interaction after accounting for the specified control variables suggests that education-related heterogeneity is not reducible to basic demographic, health, or income differences alone. This interpretation aligns with prior evidence that resource conditions shape reliance on insurance as a safeguard against future vulnerability (11, 26).

SHS also conditions the AR–PI relationship in Malaysia, with better self-rated health strengthening the association (H3b supported). This finding contrasts with studies linking poorer subjective health to greater insurance demand (20, 26). A plausible interpretation is that better self-rated health makes preventive insurance planning feel more feasible and timely, whereas poorer self-rated health may increase perceived need while reducing perceived affordability, insurability, or readiness to act. Under this interpretation, SHS differentiates how actionable AR becomes rather than simply indicating the perceived desirability of protection. Future research should directly measure perceived insurability, affordability, and planning efficacy to clarify this pathway.

Study 3 refines the country-specific findings by showing that national context matters selectively rather than uniformly. The absence of a country-conditioned AR–PI association suggests that the basic expectancy-based link between aging-related uncertainty and PMI intention is not confined to one national setting. In both China and Malaysia, AR appears to operate as a broadly salient future-oriented concern. However, the role of education as a boundary condition is more context dependent. The education-related heterogeneity observed in Malaysia but not in China suggests that educational differences become more behaviorally visible when consumers face different opportunity structures for interpreting, comparing, or acting on private insurance options. In this sense, education should not be treated as a universally stable moderator of risk-driven insurance intention; its role depends on how national healthcare and insurance arrangements shape the perceived instrumentality and substitutability of PMI. By contrast, the absence of country-conditioned SHS moderation suggests that health-related perceived actionability may be less sensitive to national context than education-related planning resources. Given the measurement-invariance results, this interpretation is best understood as boundary-condition evidence from the pooled model rather than as a formal comparison of country-specific latent path coefficients.

Taken together, the findings clarify how aging-related uncertainty, individual resources, and national context jointly shape PMI intentions among young consumers. AR emerges as a robust correlate of PMI intention, while education and subjective health function primarily as boundary conditions rather than direct predictors. The results also qualify a simple resource-based explanation: individual resources do not shape risk-to-intention translation in isolation, but operate within institutional and market environments that affect whether PMI is perceived as a salient and actionable response. Future research should therefore directly measure the informational, economic, and motivational processes through which consumers convert aging-related risk perceptions into insurance planning.

### 5.2 Limitations and future research

Despite its contributions, this study has several boundary conditions that motivate future research. First, the cross-sectional design supports theory-consistent associations but limits causal inference; future studies could employ longitudinal, time-lagged, or experimental designs to better establish temporal ordering and reduce reverse-causality concerns. Second, the sample was drawn from young consumers in major metropolitan areas with relatively developed insurance markets via purposive online recruitment; future research should extend geographic coverage and use probability-based or stratified sampling to assess broader population generalizability. Third, EDU and SHS were modeled as single-item demographic indicators, which is common for categorical education and global self-rated health but may attenuate interaction effects; future work could adopt richer operationalizations (e.g., health scales, health literacy, or education-related financial knowledge). Finally, because partial measurement invariance was not established for several key constructs, the pooled country-related effects should be interpreted as boundary-condition evidence rather than formal MGA-style cross-national path comparisons; future research should refine the measurement instrument to strengthen cross-group comparability.

### 5.3 Practical implications

This study provides practical implications for insurers, policymakers, and healthcare stakeholders seeking to promote informed PMI uptake in aging societies. The consistently strong association between AR and PMI intentions suggests that aging-related uncertainty can serve as a common communication anchor across contexts. Insurers can frame PMI as a forward-looking planning tool for later-life healthcare exposure, using concrete scenarios such as rising out-of-pocket medical costs, reimbursement gaps, long-term treatment needs, and the financial burden of care in later life.

The Malaysia findings suggest that product design and communication should prioritize reducing comprehension and affordability barriers. Because the AR–PI link is stronger among lower-EDU consumers, insurers should avoid complex benefit language and instead provide plain-language benefit summaries, standardized one-page key facts statements, premium-to-benefit examples, and simple claim illustrations showing what is covered, what is excluded, and how reimbursement works. Basic supplemental PMI options with transparent exclusions, flexible premium payment schedules, and employer- or community-based group arrangements may also reduce practical adoption barriers. For consumers reporting better SHS, communication can position PMI as preventive financial planning to be considered before health deterioration increases perceived difficulty, exclusions, or waiting-period concerns.

The China findings imply a different practical emphasis. Because EDU and SHS do not significantly differentiate the AR–PI association in the country-specific model, PMI communication may be more effective when framed around shared aging-related uncertainty and the complementary role of private coverage rather than narrowly segmented by education or self-rated health. Insurers can make this complementarity concrete by showing how PMI addresses residual exposure after public reimbursement, such as deductibles, co-payments, non-listed medicines, higher-tier hospital services, or services outside public catalogues. Policymakers can support informed uptake by standardizing benefit labels, clarifying the functional boundary between public and private protection, and improving reimbursement coordination so that consumers can more easily understand where PMI adds value.

The pooled boundary-condition evidence further suggests that education-based segmentation should not be transferred mechanically across markets. A common AR-based message may be broadly useful in both China and Malaysia, but the way this message is translated into product design, explanation, and distribution should reflect national insurance arrangements. In Malaysia, education-sensitive simplification and affordability support appear especially important; in China, clearer public–private complementarity and standardized “top-up” functions may be more salient. Thus, effective PMI promotion in aging societies requires a dual strategy: a shared risk-based framing that makes later-life uncertainty salient, combined with context-specific implementation that helps consumers see PMI as understandable, affordable, and functionally complementary to existing protection.

### 5.4 Conclusion

This study examined how perceived aging risk (AR) is associated with private medical insurance purchase intentions (PI) among adult consumers aged 18-64 and whether the AR–PI association is conditioned by education (EDU), subjective health status (SHS), and national context. The findings show that AR is consistently and positively associated with PI, confirming the relevance of aging-related risk perceptions for PMI purchase intentions in both China and Malaysia.

The study contributes to an expectancy-based account of PMI purchase intentions by showing that AR is a salient future-oriented risk appraisal linked to PI. It also refines a resource-based account of heterogeneity by showing that EDU and SHS are not uniform direct predictors of PI, but operate as boundary conditions in the AR–PI association. The China–Malaysia comparison further indicates that national context matters primarily for education-related heterogeneity, rather than for the overall AR–PI association. These findings suggest that PMI promotion in aging societies should use AR-based communication as a common foundation, while adapting product explanation, consumer segmentation, and public–private insurance coordination to local institutional contexts.

## Data Availability

The original contributions presented in the study are included in the article/Supplementary material, further inquiries can be directed to the corresponding author.
