# Supplementary material for: Perceived aging risk and private medical insurance intentions: education, subjective health, and a China–Malaysia comparison
Source: Front Public Health. 2026 Jun 4;14:1731431. doi: 10.3389/fpubh.2026.1731431 (PMC13275438; doi:10.3389/fpubh.2026.1731431)
Supplement: Supplementary file 1 [file Supplementary_file_1.pdf]

Appendix A: Results of Measurement Invariance Testing (MICOM: Measurement Invariance of Composite Models)

**Categorical Variable**	**Constructs**	**Configurational Invariance (Step 1)**	**Compositional Invariance (Step 2)**			**Partial Measurement Invariance**
			**Original Correlation**	**5.00 %**	**Permutation p value**	
Country (CTY)	AR	Yes	0.999	1.000	0.000	**No**
	PI	Yes	1.000	1.000	0.047	**No**
	EDU	Yes	1.000	1.000	0.148	Yes
	SHS	Yes	1.000	1.000	0.000	**No**

Note: Step 1: Normally, this is automatically established. Step 2: Check whether the original correlation is higher than 5% and the permutation p-value is higher than 0.05.

Appendix B: Questionnaire measurement items

Constructs	Items
**Aging Risk (AR)**	**AR1:** I think the increasing aging is not a good thing for me personally.<br>**AR2:** I believe that increasing aging increases the future risks of elderly care for myself and important individuals in my life.<br>**AR3:** I believe that increasing aging increases the future risks of income reduction, like unemployment and delayed retirement, for myself and important individuals in my life.<br>**AR4:** I believe that increasing aging brings about higher future burden for the social insurance, and future medical cost risks for myself and important individuals in my life.<br>**AR5:** I believe that the risks associated with aging described in the above questions are highly likely to occur in the future.<br>**AR6:** I am very worried about being affected by the future risks associated with aging as described in the questions above.<br>**AR7:** I consider the future risks associated with aging to be a serious matter that demands attention.<br>**AR8:** I am concerned about aging-related social phenomenon and issues, and am often anxious about the future.
**Private medical insurance purchase intentions (PI)**	**PI1:** I intend to purchase medical insurance in the near future.<br>**PI2:** I plan to purchase medical insurance in the near future.<br>**PI3:** I am likely to purchase medical insurance in the near future.<br>**PI4:** I will try to purchase medical insurance.<br>**PI5:** I expect to purchase medical insurance.<br>**PI6:** I want to purchase medical insurance.

Appendix C: Cross-Loadings Results of 3 Studies

	Study 1		Study 2		Study 3	
	**AR**	**PI**	**AR**	**PI**	**AR**	**PI**
**AR1**	0.737	0.537	0.782	0.745	0.793	0.695
**AR2**	0.817	0.607	0.869	0.644	0.862	0.676
**AR3**	0.808	0.641	0.827	0.598	0.846	0.679
**AR4**	0.814	0.64	0.833	0.561	0.849	0.66
**AR5**	0.816	0.622	0.821	0.638	0.84	0.679
**AR6**	0.796	0.608	0.841	0.572	0.847	0.658
**AR7**	0.795	0.634	0.847	0.571	0.854	0.678
**AR8**	0.743	0.628	0.799	0.601	0.79	0.655
**PI1**	0.677	0.862	0.673	0.878	0.728	0.892
**PI2**	0.66	0.867	0.634	0.86	0.703	0.886
**PI3**	0.668	0.86	0.622	0.852	0.706	0.882
**PI4**	0.688	0.862	0.587	0.847	0.702	0.884
**PI5**	0.657	0.838	0.689	0.852	0.716	0.864
**PI6**	0.665	0.869	0.658	0.858	0.695	0.871

Note: All indicators loaded higher on their intended constructs than on the alternative construct across the three studies, supporting discriminant validity at the item level.
